# Comparative analysis of tuberculin and defined antigen skin tests for detection of bovine tuberculosis in buffaloes (*Bubalus bubalis*) in Haryana state, India

**DOI:** 10.1186/s12917-024-03913-3

**Published:** 2024-02-23

**Authors:** Mohit Kumar, Tarun Kumar, Babu Lal Jangir, Mahavir Singh, Devan Arora, Yogesh Bangar, Andrew Conlan, Martin Vordermeier, Douwe Bakker, S. M. Byregowda, Sreenidhi Srinivasan, Vivek Kapur, Naresh Jindal

**Affiliations:** 1 Department of Veterinary Public Health and Epidemiology, Lala Lajpat Rai University of Veterinary and Animal Sciences, Hisar, 125 004 India; 2 Veterinary Clinical Complex, Lala Lajpat Rai University of Veterinary and Animal Sciences, Hisar, 125 004 India; 3 Department of Veterinary Pathology, Lala Lajpat Rai University of Veterinary and Animal Sciences, Hisar, 125 004 India; 4 College Central Laboratory, Lala Lajpat Rai University of Veterinary and Animal Sciences, Hisar, 125 004 India; 5 Regional Centre at Karnal, Lala Lajpat Rai University of Veterinary and Animal Sciences, Hisar, 125 004 India; 6 Department of Animal Genetics and Breeding, Lala Lajpat Rai University of Veterinary and Animal Sciences, Hisar, 125 004 India; 7 Disease Dynamics Unit, Department of Veterinary Medicine, University of Cambridge, Cambridge, UK; 8 Animal and Plant Health Agency, Surrey, UK; 9 Technical Consultant and Independent Researcher, Lelystad, The Netherlands; 10 Institute of Animal Health and Veterinary Biologicals, Bengaluru, India; 11 Department of Animal Science, The Pennsylvania State University, University Park, PA USA; 12 The Huck Institutes of the Life Sciences, The Pennsylvania State University, University Park, PA USA

**Keywords:** Bovine tuberculosis, Buffaloes, Haryana, India, SIT, SICCT, DST

## Abstract

**Background:**

Bovine tuberculosis (bTB) is a chronic disease that results from infection with any member of the *Mycobacterium tuberculosis* complex. Infected animals are typically diagnosed with tuberculin-based intradermal skin tests according to World Organization of Animal Health which are presently in use. However, tuberculin is not suitable for use in BCG-vaccinated animals due to a high rate of false-positive reactions. Peptide-based defined skin test (DST) antigens have been identified using antigens (ESAT-6, CFP-10 and Rv3615c) which are absent from BCG, but their performance in buffaloes remains unknown. To assess the comparative performance of DST with the tuberculin-based single intradermal test (SIT) and the single intradermal comparative cervical test (SICCT), we screened 543 female buffaloes from 49 organized dairy farms in two districts of Haryana state in India.

**Results:**

We found that 37 (7%), 4 (1%) and 18 (3%) buffaloes were reactors with the SIT, SICCT and DST tests, respectively. Of the 37 SIT reactors, four were positive with SICCT and 12 were positive with the DST. The results show that none of the animals tested positive with all three tests, and 6 DST positive animals were SIT negative. Together, a total of 43 animals were reactors with SIT, DST, or both, and the two assays showed moderate agreement (Cohen’s Kappa 0.41; 95% Confidence Interval (CI): 0.23, 0.59). In contrast, only slight agreement (Cohen’s Kappa 0.18; 95% CI: 0.02, 0.34) was observed between SIT and SICCT. Using a Bayesian latent class model, we estimated test specificities of 96.5% (95% CI, 92–99%), 99.7% (95% CI: 98–100%) and 99.0% (95% CI: 97–100%) for SIT, SICCT and DST, respectively, but considerably lower sensitivities of 58% (95% CI: 35–87%), 9% (95% CI: 3–21%), and 34% (95% CI: 18–55%) albeit with broad and overlapping credible intervals.

**Conclusion:**

Taken together, our investigation suggests that DST has a test specificity comparable with SICCT, and sensitivity intermediate between SIT and SICCT for the identification of buffaloes suspected of tuberculosis. Our study highlights an urgent need for future well-powered trials with detailed necropsy, with immunological and microbiological profiling of reactor and non-reactor animals to better define the underlying factors for the large observed discrepancies in assay performance, particularly between SIT and SICCT.

**Supplementary Information:**

The online version contains supplementary material available at 10.1186/s12917-024-03913-3.

## Introduction

Bovine tuberculosis (bTB) is a chronic disease of cattle caused by members of the *Mycobacterium tuberculosis* complex (MTBC). It is a multi-host disease that infects a diverse group of domesticated and wild animals. In cattle, bTB negatively affects milk production and fertility, thus leading to economic losses [1–3]. Importantly, bTB is a neglected zoonotic disease that crosses the species barrier and can infect humans, with the major routes of transmission being consumption of unpasteurized milk or undercooked meat [4].

Tuberculin-based intradermal skin test, recommended by the World Organization for Animal Health (WOAH), is currently used for screening of animals for bTB [5]. Tuberculin skin testing is based on a delayed type hypersensitivity to purified protein derivatives (PPDs) from standard cultures of *Mycobacterium avium* (PPD-A) and *Mycobacterium bovis* (PPD-B). The single intradermal test (SIT) involves PPD-B alone, while the single intradermal comparative cervical test (SICCT) utilizes with both PPD-B and PPD-A [5]. Importantly, the presence of cross-reactive antigens between field and vaccine strains leads to unacceptably high rates of false-positive reactions from tuberculin-based diagnostics in Bacille Calmette-Guérin (BCG)–vaccinated animals and necessitates new diagnostics to provide the ability to differentiate infected from vaccinated animals (DIVA) [6]. This limits opportunities for the development and implementation of BCG vaccination-based control programs to help accelerate the control of bTB.

We tested female buffaloes in organized dairy farms in two districts of Haryana, India. The WOAH-recommended interpretations of the standard tuberculin-based tests were used alongside peptide-based defined skin test (DST) antigens. The DST antigens comprise of ESAT-6, CFP-10 and Rv3615c, that have been recently shown to have DIVA potential [6–8].

Systematic evaluation of performance of diagnostic tests for bovine tuberculosis is hampered by the lack of a proper gold standard for identification of infected animals [5]. The Walter-Hui latent class model provides a theoretical framework to address this problem, allowing the sensitivity and specificity of a set of competing diagnostic tests to be estimated when samples are available from at least two populations with differing prevalence [9, 10]. In recent years this approach has been used to evaluate the relative performance of bTB diagnostics in dairy cattle using field data from Ireland, Spain, France, Northern Ireland, Brazil, and Egypt [11–19]. This approach has also been used for evaluation of serological test for diagnosis of brucellosis in buffaloes in Pakistan [20]. No systematic performance of bTB diagnostics in buffalo has been carried out in India. The aim of this study was to evaluate DST with WOAH recommended tuberculin test in buffaloes. We use the foundational Walter-Hui latent class model to provide first estimates of the relative sensitivity and specificity of the SIT, SICCT and DST tests in buffaloes with a view to assessing the performance of the novel DST test with respect to the two international standards.

## Materials and methods

### Study population

Haryana, a state in Northern India, is located between 27° 37′ to 30° 35′ latitude and 74° 28′ to 77° 36′ longitude. Based on agro-climatic zones in India, Haryana is in Zone-VI (Trans-Gangetic Plains Region). Geographically, the state is further subdivided into two zones i.e., Eastern and Western. To account for this, a total of 49 farms were identified in two districts within the State: District A from the western zone and district B from the eastern zone for a prospective study to compare performance of PPDs and DST to detect bTB infection in buffaloes. A total of 543 female buffaloes (326 in district A and 217 in district B) from these organized dairy farms were included in this study, based on an assumed prevalence of 15% in female buffaloes at 20% precision (95% confidence interval). The animals were stratified into three age groups: calves (6 months–1 year of age), heifers (1–3 years of age) and adults (more than 3 years of age). Calves less than 6 months of age and adult buffaloes in an advanced stage of pregnancy or who had recently calved were excluded from this study. Written consent was obtained from the dairy owners before their inclusion in the study. They were apprised about bovine tuberculosis, its clinical findings, importance, zoonotic nature, testing and its benefits, and possible risks. The animals were tested and the dairy owners were advised to keep reactor animals in isolation and seek the advice of their veterinarians for specific guidance.

### Skin testing

The intradermal skin test was performed on both sides of the neck. On the left side of the neck, 0.1 ml each of bovine PPD (strain AN5; PPD-B; 3000 IU) and avian PPD (strain D4ER; PPD-A; 2500 IU) (Prionics, Switzerland) were administered intradermally using McLintock syringes (Bar Knight McLintock Limited, Scotland). On the right side of the neck, the peptide-based DST was injected. The DST contained chemically synthesized peptides representing ESAT6, CFP10 and Rv3615c prepared at > 98% purity with a final concentration of 20 µg/peptide [21]. The ready-to-use DST cocktail (synthesized by GenScript, USA and USV Private Limited, India) was reconstituted to achieve a final concentration of 20 µg for each individual peptide in a total volume of 0.1 ml at the time of administration. Before administration, skin thickness was measured in millimeters (0-h value) using a vernier caliper. Skin thickness was measured again at 72 ± 4 h by the same operator. The difference in skin thickness (72 h–0 h) was determined, and animals with an increase in skin thickness of 4 mm or more due to bovine PPD (Single intradermal test; SIT) or PPD-B minus PPD-A (Single intradermal comparative cervical tuberculin test; SICCT) were classified as reactors. For the DST, animals with increase in skin thickness by 2 mm were considered as reactors. The cross-classified results of the 3 tests in the 19 study herds are presented in Supplementary Table 1.

### Statistical analyses

The agreement between SIT, SICCT and DST was estimated using Cohen’s Kappa [22]. The Walter-Hui latent class model was implemented in stan, estimated by Hamiltonian Markov Chain Monte Carlo (MCMC) and analyzed in R using the rstan package [23, 24]. Convergence was assessed through visual inspection of the chains and standard diagnostic statistics ($\widehat{R}=1$ for all parameters after $2,000$ iterations for 8 chains). Estimated parameters are presented as median posterior values with 95% Bayesian credible intervals (CrI).

The key assumption of the Walter-Hui (WH) model is conditional independence between tests, i.e., the probability of a test $k$ being positive for individual ($i$), $P\left({T}_{i,k}=1\right)$ only depends on the latent (in this case true) disease status of the individual ($D\in \{0,1\}$) and not the response of the other tests. Under this assumption the (conditional) probability of a positive test result given that an animal is infected ($D=1$) or disease free ($D=0$) can then be modelled by a single parameter for each test:

$$\begin{array}{c}P\left({T}_{i,k}=1|D=1\right)={a}_{k}\\ P\left({T}_{i,k}=1|D=0\right)={b}_{k}\end{array}$$

and the sensitivity of test $k$ will then simply be ${a}_{k}$ and the specificity will be $1-{b}_{k}$.

Following (1, 30), and to allow for an extension to model any conditional dependence between tests, we parameterised the model using a probit ($\Phi$) link function:

$$\begin{array}{c}P\left({T}_{i,k}=1|D=1\right)=\Phi \left({a}_{k,1}\right)\\ P\left({T}_{i,k}=1|D=0\right)=\Phi \left({a}_{k,0}\right)\end{array}$$

To ensure numerical stability we restrict the sensitivity parameters (on the probit scale) ${a}_{k,1}$ to the range $\left[-8,8\right]$. A common issue with this class of models is that the likelihood can be symmetric under relabeling of the latent variable. This leads to a multi-modal posterior distribution where two modes can provide an equivalent fit to the data corresponding to the situation where the true positive rate is greater than the false positive rate (TPR > FPR, $\Phi \left({a}_{k,1}\right)> \Phi \left({a}_{k,0}\right)$ and the inverse where the FPR > TPR ($\Phi \left({a}_{k,0}\right)> \Phi \left({a}_{k,1}\right))$. Given the performance of the SIT and SICCT tests in other contexts we consider the situation where the FPR > TPR to be biologically implausible.

To force identifiability of the model (and avoid a label switching problem during estimation) we place a prior restriction on the range of parameters such that no tests can have a specificity of $<84\mathrm{\%}$ which corresponds to restricting ${a}_{k,0}$ to the half-range $\left[-8,-1\right]$. We otherwise use uninformative normal (mean = 0, sd = 1) priors for ${a}_{k,0}$ and ${a}_{k,1}$ and a beta (1,1) prior for the true prevalence within each herd (the latent variable).

The SIT and SICTT test results have an implicit dependence on each other in the sense that they are both calculated based on the magnitude of the observed reaction to bovine tuberculin. The comparison to avian tuberculin in the SICCT test is intended to raise specificity and reduce sensitivity. A biological dependence between the tests – implies - but does not guarantee that there will be a statistical association between the results of the two tests strong enough to violate the assumption of conditional independence. If the correlation between (true) infection status and the results of each test result is large compared to the correlation between the test results themselves then the conditional dependence will not affect the parameter estimates and the test results can effectively be treated as (statistically) independent.

Dendukuri et al. [25] showed how the the pairwise probability of agreement between each pair of diagnostic tests ($k,k\mathrm{^\prime}$):

$${\alpha }_{k,k\mathrm{^\prime}}=\frac{\sum_{i=1}^{N}{T}_{i,k}{T}_{i,k\mathrm{^\prime}}-\left(1-{T}_{i,k}\right)\left(1-{T}_{i,k\mathrm{^\prime}}\right)}{N}$$

can be used to assess model fit and test the assumption of conditional independence. Any systematic differences between the observed (${\alpha }_{k,k\mathrm{^\prime}}$) and expected values from the estimated model (${\alpha }_{k,k\mathrm{^\prime}}^{*}$) would imply a violation of the assumption of conditional independence. We can use draws from the posterior predictive distribution of ${\alpha }_{k,k\mathrm{^\prime}}^{*}$ for our fitted model to form a posterior predictive *p*-value [26]:

$$P\left({\alpha }_{k,k\mathrm{^{\prime}}}^{*}>{\alpha }_{k,k\mathrm{^{\prime}}}\right)$$

If the model fits well, the value of $P\left({\alpha }_{k,k\mathrm{^\prime}}^{*}>{\alpha }_{k,k\mathrm{^\prime}}\right)$ is expected to be close to $0.5$, with extreme values close to $0$ or $1$ indicating a lack of fit (i.e., < 0.05 or > 0.95).

To further explore the potential for conditional dependence between the SIT and SICCT tests we suggest an alternative model that models this dependence through assuming that that the sensitivity and specificity of these tests can be interpreted as points on shared receiver-operator characteristic (ROC) curve. This is equivalent to reparametrizing the WH model to introduce an assumed functional form that links the parameters for these two tests.

For the functional form we used the normal model of a ROC curve [27] which arises from assuming that the false and true positive rates for each tests have equal variance ${\sigma }_{g}$. The parameter ${\sigma }_{g}$ determines the shape of the ROC curve and the relationship between the sensitivity and specificity for a group of tests $g$. Using this functional form, we can write the conditional probabilities of a positive result as:

$$\begin{array}{c}P\left({T}_{i,k}=1|D=0\right)=\Phi \left({a}_{k,0}\right)\\ P\left({T}_{i,k}=1|D=0\right)=\Phi ({\Phi }^{-1}\left({a}_{k,0}\right)+{i}_{g}{\sigma }_{g})\end{array}$$

In this case we have two groups $g=\mathrm{1,2}$ with a shared parameter ${\sigma }_{1}$ constraining the parameters for the SIT and SICCT tests to lie on a shared ROC curve and a separate parameter ${\sigma }_{2}$ for the DST. For brevity, we will thus refer to this as the WHROC model.

For the WHROC model we restricted the range of $${\sigma }_{g}$$ to [0,8] and used an indicator variable $${i}_{g}$$ to choose between the default (TPR > FPR, $${i}_{g}=1)$$ and alternative (FPR > TPR, $${i}_{g}=1)$$ prior assumptions. As well as allowing us to explicitly model dependence between the SIT and SICCT tests, this formulation allows us to illustrate how parameter estimates change for the alternative prior assumption (which as above we consider to be biologically unfeasible).

Thus, we fitted a total of three models corresponding to the baseline WH model and the WHROC model with both the default (WHROC) and alternative prior (WHROC2) assumptions. To compare the estimated models, we used leave-one-out (LOO) cross-validation [28]. The expected log pointwise predictive density: $\widehat{elpd}$, which measures the predictive accuracy of the model when a single observation is dropped out, was estimated by Pareto smoothed importance sampling (PSIS-LOO). The difference $\widehat{\Delta elpd}$ between $\widehat{elpd}$ for alternative models fitted to the same data provides a measure of their relative predictive accuracy. The standard error on the difference gives a measure of uncertainty. Standard errors (sd) comparable to the magnitude of the difference $\widehat{\Delta elpd}$ suggest that the relative predictive accuracy of the two models is indistinguishable.

## Results

**Fig. 1:**
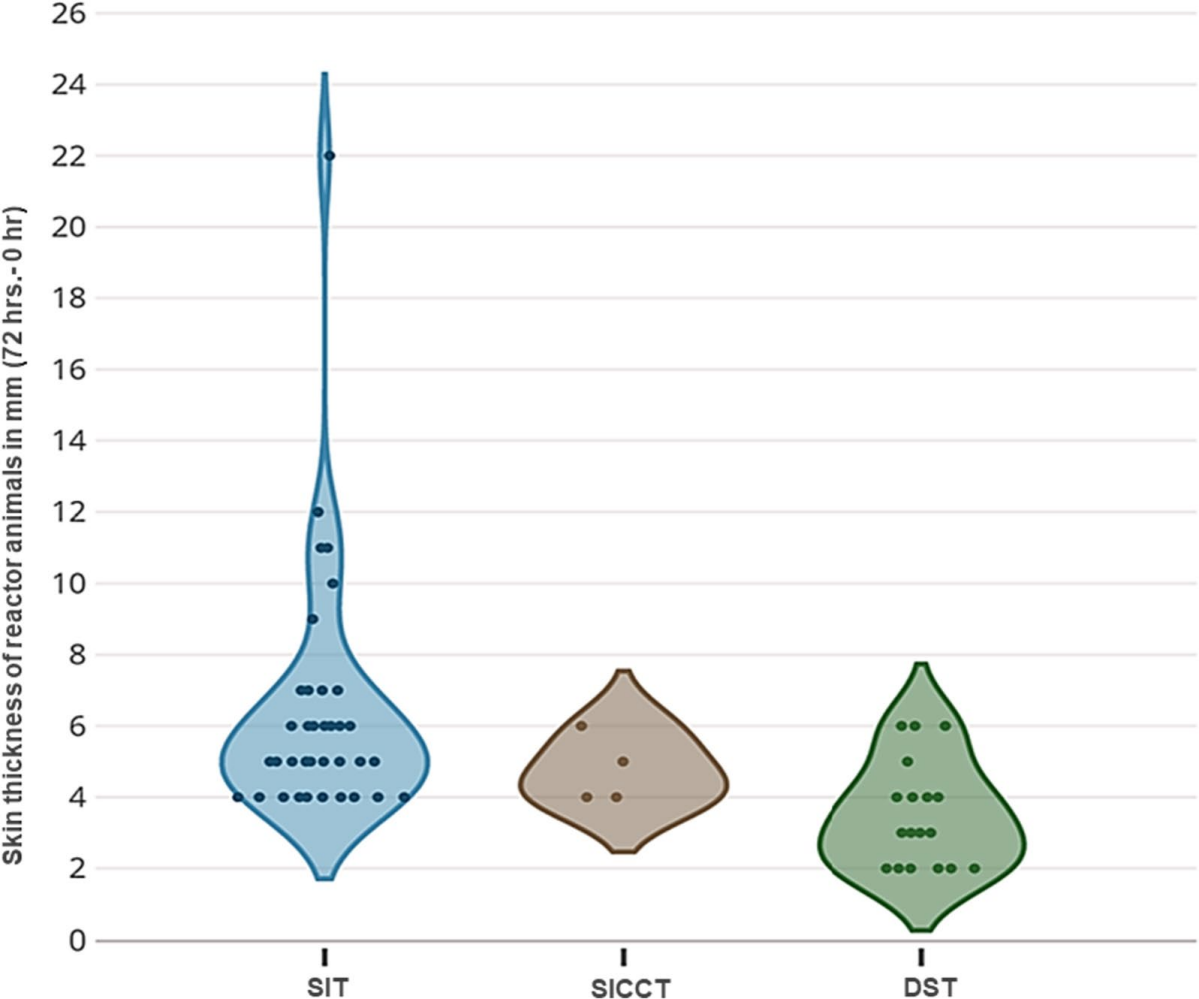
Distribution of skin thickness amongst the 43 buffaloes identified as reactors by different skin tests. SIT, Single intradermal test; SICCT, Single intradermal comparative cervical test; DST, Defined skin test

Out of 543 female buffaloes screened for bTB in 49 organized dairy farms, 37 (6.8%) animals in both districts were found to be reactors by SIT (Fig. 1). Only 4 (< 1%) animals were found reactors with the SICCT test; three of which did not show any response to PPD-A. By DST, 18 (3%) buffaloes were found to be reactors as per the cut-off of $\ge$ 2 mm (Fig. 1). Swelling at the site of administration of PPDs or DST in positive cases was observed. Considering SIT alone, 30 and 7 buffaloes were reactors in district A and B, respectively. Of the 30 reactor animals identified in district A, 21 were adult animals while eight were heifers and one was a calf. In district B, all seven reactors identified by SIT were adults. Of the 37 reactors identified by SIT, 23 (62%) were milch animals. Of the DST positive animals, 16 (12 adults, 2 heifers, and 2 calves) were in district A while two (both adults) were in district B. Of the SICCT positive animals, three were adults and one was heifer and all were from district A. Of the DST reactors, it was observed that six animals were negative by SIT. Out of the 49 dairy farms whose animals were tested, reactor animals by at least one of the tests used were identified in only 18 dairy farms. None of the tested animals in the remaining 31 dairy farms showed reactivity to tuberculin or DST.

The result showed that 4 of the 37 SIT responders did not show a measurable response to PPD-A, while 3 of the 4 SICCT positive animals were non-responders to PPD-A. Of the 37 SIT responders, 14 animals had higher PPD-A response than PPD-B. Forty-five animals had a skin thickness difference of 2–3 mm by SIT; these animals were categorized as inconclusive reactors. The data were also analyzed with respect to the magnitude of skin thickness seen at 72 h post-administration of antigens. With bovine PPD alone, 27 animals had differences in skin thickness between 4–6 mm while in the remaining 10 animals the difference was more than 7 mm. Using SICCT, all four reactors had 4–6 mm difference in skin thickness. With DST, 10 buffaloes were in the range of 2–3 mm and 8 buffaloes showed 4–6 mm increase in skin thickness.

**Fig. 2:**
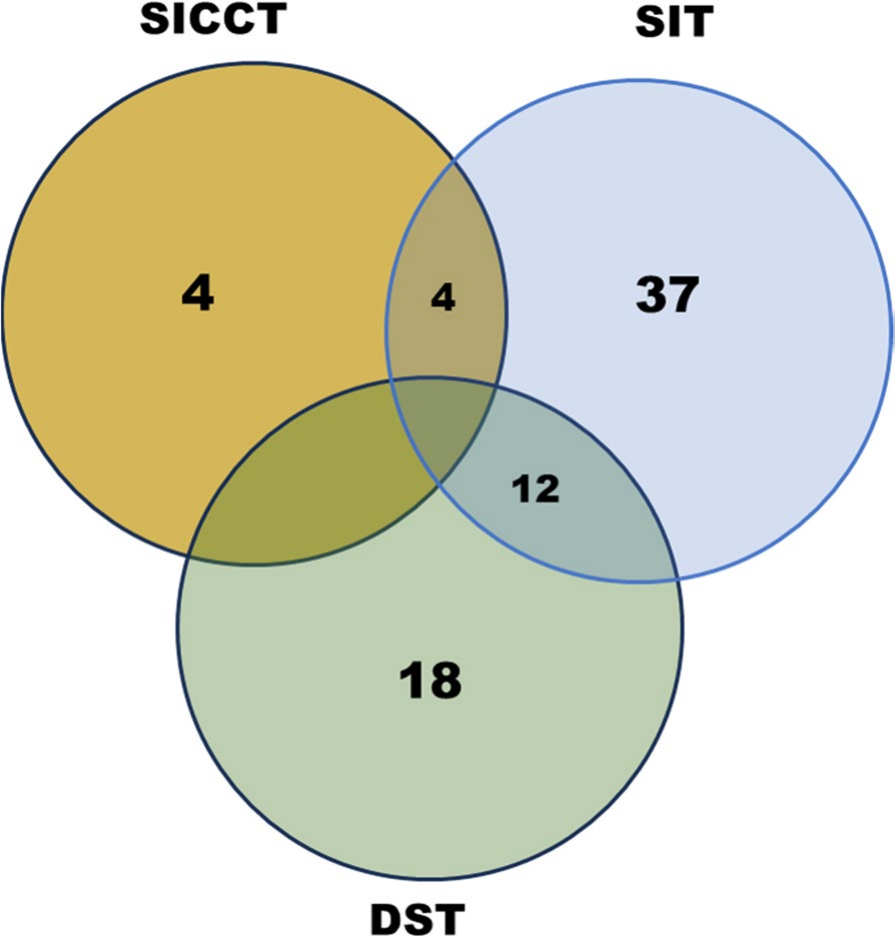
Number of adult buffaloes showing reaction to bovine and avian tuberculins and defined antigen skin test. SIT, Single intradermal test; SICCT, Single intradermal comparative cervical test; DST, Defined skin test

The study identified several discrepancies in reactor status based on the test (Fig. 2). Twenty-five animals classified as reactors by SIT were negative by DST (Fig. 2). None of the animals tested was classified as a reactor by both SICCT and DST. A total of 6 animals that were DST positive but SIT negative. Considering both SIT and DST, 43 animals were found to be reactors. Correlation analyses revealed that SIT and DST showed moderate agreement with a Cohen’s Kappa of 0.41 (95% CI: 0.23, 0.59) for test positive cases (Table 1); whereas, there was a low Kappa agreement of 0.18 (95% CI: 0.02, 0.34 was found between SIT and SICCT.

**Table 1:** Agreement of SIT with SICCT and DST

**Test**	**SICCT**		**DST**	
	**Negative**	**Positive**	**Negative**	**Positive**
SIT negative	506	0	500	06
SIT positive	33	04	25	12
Total	539	04	525	18
Cohen’s Kappa (95% CI)	0.18 (0.02, 0.34), *p* = 0.001		0.41 (0.23, 0.59), *p* = 0.001	

*SIT* Single intradermal test, *SICCT* Single intradermal comparative cervical test, *DST* Defined skin test

**Fig. 3:**
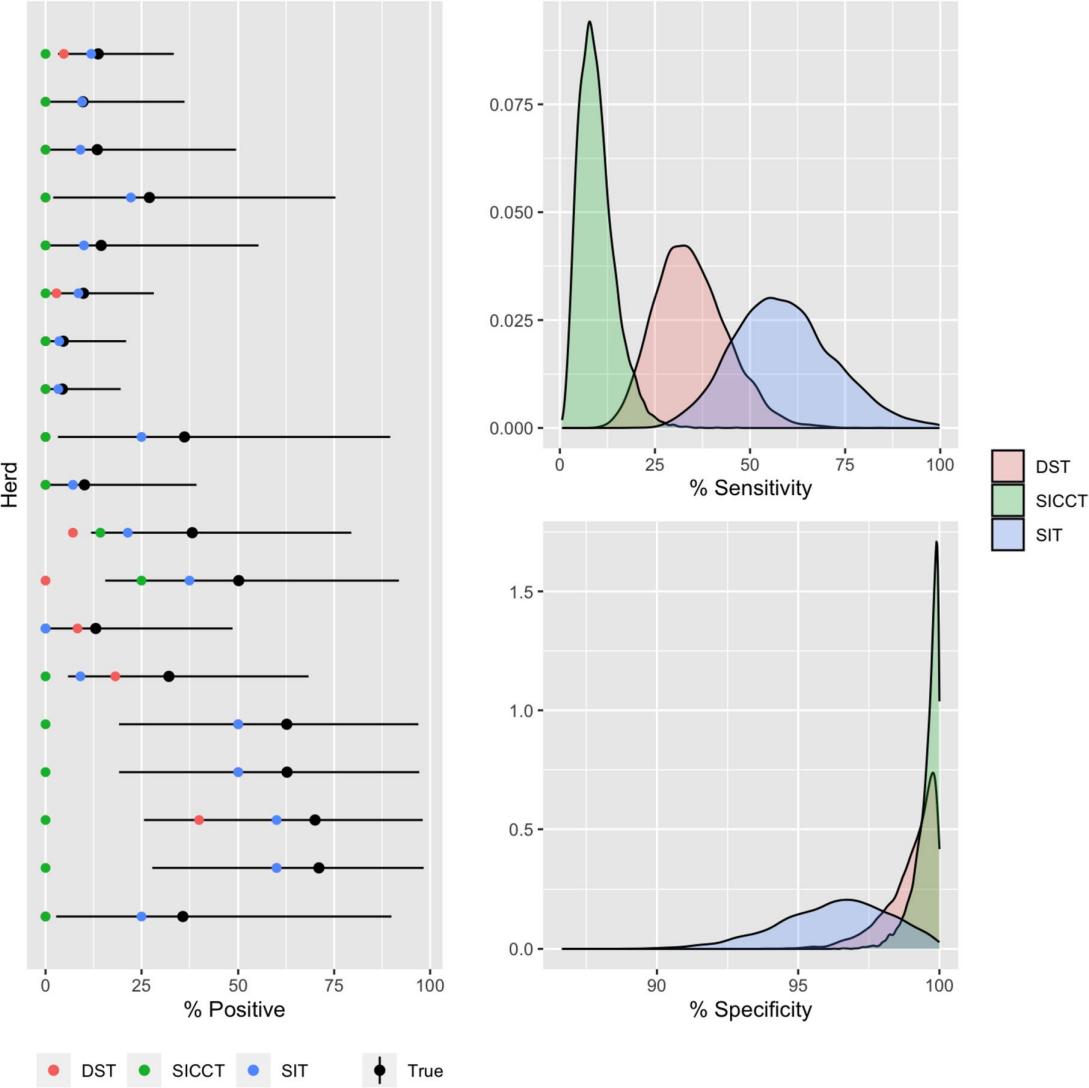
(Left) Posterior estimates of the true within-herd prevalence (Black points with lines indicate 95% CrI), plotted against observed reactor status as measured by the DST (red), SICCT (green) and SIT (blue) tests. (Right) Posterior distributions for the sensitivity and specificity of the SIT, SICCT and DST diagnostic tests from the baseline WH model

The three fitted latent class models all demonstrate an agreement with the apparent reactor status across all infected and uninfected herds (Supplementary Table 1). The entire observed values lie within the 95% posterior predictive intervals of the estimated model (Fig. 3) and are indistinguishable in terms of their fit as measured by LOO cross validation (Table 2). Posterior predictive *p*-values - based on the pairwise probability of agreement between each pair of diagnostic tests - are all within a 95% interval with no evidence for conditional dependence between the tests for all three models (Table 3). Thus, based on this data we have no evidence to suggest there is a statistical dependence between any of the tests and focus on reporting estimates from the baseline WH model. Estimates from the WHROC model have almost completely overlapping posteriors, with equivalent point estimates for the sensitivity and specificity of the SICCT test, but slightly lower point estimates for the DST and SIT respectively (Table 4). Using the alternative prior assumption for the WHROC2 model (FPR > TPR), which we consider biologically unfeasible, estimates of the true prevalence are reflected – with the WHROC2 model estimating a much higher prevalence of infection overall compared to WHROC. For this alternative model all three tests have an estimated sensitivity close to zero and the observed apparent prevalence is explained entirely by variation in the estimated specificity. Given the lack of statistical support for conditional dependence between the three tests we would argue that the only benefit of the WHROC model in this case is providing a more elegant way to specify a prior distribution to avoid the unfeasible posterior mode selected by the WHROC2 model.

**Table 2:** Model comparison through leave-one-out (loo) cross validation

**Model**	$\widehat{\boldsymbol{\Delta }{\varvec{e}}{\varvec{l}}{\varvec{p}}{\varvec{d}}}$	**sd**
WH	0.0	
WHROC2	-0.5	1.2
WHROC	-1.7	2.0

*WH* Walter-Hui, *WHROC* Walter-Hui receiver-operator characteristic

**Table 3:** Posterior predictive p-values for pairwise probability of agreement between tests

**Model**	**Interaction**	**Posterior predictive *p*-value**
WH	SIT-SICCT	0.73
	SIT-DST	0.74
	SICCT-DST	0.83
WHROC	SIT-SICCT	0.62
	SIT-DST	0.65
	SICCT-DST	0.7
WHROC2	SIT-SICCT	0.66
	SIT-DST	0.69
	SICCT-DST	0.72

*WH* Walter-Hui, *WHROC* Walter-Hui receiver-operator characteristic, *SIT* Single intradermal test, *SICCT* Single intradermal comparative cervical test, *DST* Defined skin test

**Table 4:** Estimated sensitivity and specificity of bTB diagnostics from latent class analysis with 95% Bayesian credible intervals (CI)

**Model**	**Test**	**Sensitivity (95% CI)**	**Specificity (95% CI)**
**WH**	SIT	58.2 (35.3–86.8)	96.5 (92.1–99.5)
	SICCT	8.99 (2.78–21.2)	99.7 (98.3–100)
	DST	33.8 (18.4–54.9)	99.3 (96.6–100)
**WHROC**	SIT	43.1 (26.2–66.1)	95.1 (91.0–98.0)
	SICCT	7.53 (2.53–17.6)	99.8 (99.1–100)
	DST	25.6 (13.8–44.6)	98.9 (96.3–99.9)
**WHROC2**	SIT	3 (0.3–7)	96.4 (92.1–99.5)
	SICCT	0.04 (0–0.4)	92.9 (83.5–97.6)
	DST	0.5 (0.01–3)	73.0 (54.9–85.4)

All estimates quoted to 3 significant figures (apart from sensitivity for WHROC (Alt) quoted to 1 significant figure)

*SIT* Single intradermal test, *SICCT* Single intradermal comparative cervical test, *DST* Defined skin test, *WH* Walter-Hui, *WHROC* Walter-Hui receiver-operator characteristic

The WH BLCM estimates distinct differences in performance between the three diagnostic tests – albeit with relatively large overlaps in the posterior distributions (Fig. 3). The results show that the DST test has lower diagnostic sensitivity (34%, 18–55 95% CrI) compared to SIT (58%, 35–87 95% CrI) but comparable specificity (99%, 97–100 95% CrI) to the SICCT test (99.7, 98–100 95% CrI) (Table 4). Taken together, the DST has an intermediate sensitivity to SIT and SICCT but with broad and overlapping predictive / credible intervals.

## Discussion

The present study was undertaken to assess the comparative performance of tuberculin skin tests with defined skin antigen for the detection of bTB in buffaloes. With a national herd estimated to be nearly 100 million animals, buffaloes are a major contributor to milk production in India, and an understanding of test performance in this major livestock species is essential in order to identify infected animals so as to develop effective control strategies for bTB in buffaloes.

We tested female buffaloes in organized dairy farms in two districts of Haryana, India using the WOAH-recommended standard SIT and SICCT skin tests. These tests have distinct features affecting result interpretation. The SIT, while highly sensitive, can yield decreased specificity due to PPD-B induced inflammatory reactions in animals sensitized with non-tuberculous mycobacteria (NTM) due to cross-reactive antigens. The SICCT, employing simultaneous bovine and avian tuberculin injections, enhances specificity, but sacrifices sensitivity. This is because of cross-reactive immune responses in animals arising from exposure to *M. bovis* antigen shared with NTMs, which can result in reduced specificity of commonly used diagnostic tests [29]. Coinfection of tuberculous animals with NTMs or infection with *M. bovis* and exposure to NTM may also contribute to lower sensitivity of SICCT. Moreover, these tuberculin antigens are unable to differentiate infection from BCG vaccination due to cross-reactive antigens.

Recent studies highlighted antigens such as ESAT-6, CFP-10, and Rv3615c, present in field strains of *M. bovis* but absent or non-immunogenic in BCG vaccine strain, may enable the detection of infected amongst BCG-vaccinated animals [6, 30–32]. For instance, Srinivasan et al. [30] demonstrated the effectiveness of DST and recombinant fusion protein incorporating these antigens in distinguishing infected from uninfected animals and underscored DSTs DIVA capability that is absent in traditional tuberculin, and the ability to chemically synthesize DSTs provides an ease of manufacture and enables rigor in quality control. This test has previously been assessed in both experimental and field trials in cross-bred cattle [30, 31]. A proof-of-concept study to evaluate DIVA capability of DST was performed in cross-bred cattle in India [6]. Recently, a pilot DST dose optimization trial was also conducted in domestic water buffaloes [21].

In this study, we skin tested 543 female buffaloes using both tuberculins and DST. Findings revealed 37 and 4 reactors in two districts by SIT and SICCT, respectively. Intriguingly, DST identified six additional reactors negative by SIT and SICCT and 25 SIT reactors as non-reactors. These discrepancies highlight the variability in diagnostic performance, potentially influenced by tuberculin quality and environmental mycobacteria prevalence and raise important questions on performance of these tests and the underlying reasons behind these discrepancies. PPDs enable early detection of bovine tuberculosis, facilitating swift intervention to safeguard animal health, and support international trade by meeting certification standards. Their key role in preemptive disease management underscores their significance in maintaining both economic viability and global health standards in the livestock industry. The use of PPDs stands as a cornerstone in the comprehensive approach to controlling and eradicating bovine tuberculosis. However, tuberculins are crude reagents that are derived from culture supernatant of *M. bovis* AN5 strain (PPD-B) quality of the antigen can vary considerably, due to lack of proper standardization [33]. However, since a single batch of PPDs from a reputable manufacturer was used to test all animals in our study, this is unlikely to play a major role in the observed differences in test results with the DST. Another source of variation long recognized is exposure to environmental mycobacteria that may confound the accurate interpretation of tuberculin-based skin test results [3, 34, 35]. Since the prevalence of environmental mycobacteria is particularly high in tropical regions [34]; the study area being a tropical area, this may play a role in the observed discrepant test results. 

Few animals in this study exhibited higher response to both bovine and avian PPDs and in some animals, PPD-A response was higher than PPD-B. It may be possible to get such a response from environmental mycobacteria. Proano-Perez et al. [36] also reported that few animals exhibited higher PPD-A response and this response decreased significantly with age. These authors suggested that *Mycobacterium avium* complex (MAC) is more prevalent in the environment than *M. bovis*, and animals are in contact with these environmental mycobacteria early in life. However, the role of such exposures in the animals screened in our studies is unknown.

Recent studies report the presence of *M. orygis* rather than *M. bovis* in cattle and/or African buffalo [37–41]. *M. orygis* has been isolated from cattle and primates in Bangladesh [41] and in India, *M. orygis* has been reported from dairy cattle and humans [40]. Since the efficacy of the DST in diagnosing infection other than *M. bovis* has yet not been established, additional studies are required to assess the performance of the DST in animals microbiologically confirmed to be infected with *M. orygis* or other members of the MTBC including *M. tuberculosis* sensu stricto associated with bTB in relevant hosts and context.

The Bayesian latent class model estimates suggest that the DST has a sensitivity that is intermediate between the SICCT and SIT test and specificity comparable to the SICCT test. The uncertainty in these estimates, due to the relatively small sample and group sizes, is reflected in overlapping posterior distributions for diagnostic parameters and wide credible intervals for the bTB infection within each herd. The sample size may also contribute to the lack of evidence for conditional dependence between the diagnostic tests, with no measurable improvement in model fit of our alternative model which explicitly models a relationship between the SIT and SICCT responses. Studies have been conducted in cattle to assess and compare the sensitivity and specificity of diagnostic tests using Bayesian approach from different geographic and epidemiological contexts [42, 43]. Based on study undertaken in 25 bTB infected cattle herds in Thailand, Singhla et al. [42] reported 95% posterior probability interval (PPI) of SIT test sensitivity and specificity ranging from 75.3 to 95.2% and 74.2 to 92.8%, respectively while the 95% PPI of IGRA assay sensitivity and specificity was 38.6 to 74.4% and 87.0 to 98.1%, respectively. In another study in cattle, Alvarez et al. [43] reported 95% PPI of SIT test sensitivity of 40.1 to 92.2%, while the specificity was high > 99% and 95% PPI of IFN-γ assay showed a high sensitivity of 89–90% and specificity of 85.7%.

As reflected in the poor Kappa agreement reported in Table 1, each of the three diagnostic tests identify slightly different populations of animals implying they are assessing distinct facets of the animal’s immune reaction to *M. bovis*, rather than detecting the presence or absence of the organism itself. Indeed, the SIT and SICCT tests are designed to be dependent on each other in the sense that the avian response is used to increase the specificity of SICCT at the expense of sensitivity. The extent to which the sensitivity and specificity of the SIT and SICCT tests trade off against each other within this particular population is difficult to assess in the absence of microbiological or pathological confirmation of infection. The triangulation we carry out here against the DST test provides some insight into this trade-off, but validation of these estimates requires further studies including necropsies of reactor animals and culture of causative pathogens to both directly address this issue and begin to understand the other discrepancies in response between these alternative diagnostic tests.

In conclusion, our study underlines the urgent need for standardized, reliable skin tests for monitoring bTB in buffaloes, given the limitations of current diagnostics. The peptide-based DST, with its high specificity and DIVA capability together with intermediate sensitivity between SIT and SICCT, holds promise for the future implementation of vaccine-based intervention strategies in LMICs, addressing a critical gap in bTB management.

### Supplementary Information

**Supplementary material 1.**

## Data Availability

The datasets generated during and/or analyzed during the current study are presented in the manuscript. Anonymized data in a digital form and full code for the Bayesian latent class analysis are available at: github.com/monkeymyshkin/BuffaloBLCM/

## Abbreviations

bTB: Bovine Tuberculosis
DST: Defined Antigen Skin Test
PPD: Purified Protein Derivative
WOAH: World Organization of Animal Health
SIT: Single Intradermal Test
SICCT: Single Intradermal Comparative Cervical Test
MTBC: Mycobacterium Tuberculosis Complex
LMICs: Lower Middle-Income Countries
DIVA: Differentiating Infected from Vaccinated Animal
IAEC: Institutional Animal Ethics Committee
NTM: Non-Tuberculous Mycobacterium
BCG: Bacille Calmette and Guerin
MAC: Mycobacterium Avium Complex
