# Supplementary material for: Comparative analysis of tuberculin and defined antigen skin tests for detection of bovine tuberculosis in buffaloes (Bubalus bubalis) in Haryana state, India
Source: BMC Vet Res. 2024 Feb 23;20:65. doi: 10.1186/s12917-024-03913-3 (PMC11308649; doi:10.1186/s12917-024-03913-3)
Supplement: Supplementary file 1 — Supplementary material 1. [file 12917_2024_3913_MOESM1_ESM.docx]

**Supplementary Table 1**: Cross-classified results of 3 tests in 19 populations (herds)

| Diagnostic Tests | | | Populations | | | | | | | | | | | | | | | | | | | |
| --- | --- | --- | --- | --- | --- | --- | --- | --- | --- | --- | --- | --- | --- | --- | --- | --- | --- | --- | --- | --- | --- | --- |
| SIT | SICCT | DST | 1 | 2 | 3 | 4 | 5 | 6 | 7 | 8 | 9 | 10 | 11 | 12 | 13 | 14 | 15 | 16 | 17 | 18 | 19 | Total |
| - | - | - | 3 | 2 | 2 | 2 | 2 | 16 | 11 | 5 | 10 | 13 | 3 | 29 | 27 | 32 | 9 | 7 | 10 | 19 | 37 | 239 |
| + | - | - | 1 | 0 | 1 | 0 | 0 | 2 | 0 | 1 | 1 | 1 | 1 | 1 | 1 | 2 | 1 | 2 | 1 | 2 | 3 | 21 |
| - | + | - | 0 | 0 | 0 | 0 | 0 | 0 | 0 | 0 | 0 | 0 | 0 | 0 | 0 | 0 | 0 | 0 | 0 | 0 | 0 | 0 |
| + | + | - | 0 | 0 | 0 | 0 | 0 | 0 | 0 | 2 | 2 | 0 | 0 | 0 | 0 | 0 | 0 | 0 | 0 | 0 | 0 | 4 |
| - | - | + | 0 | 0 | 0 | 0 | 0 | 4 | 1 | 0 | 1 | 0 | 0 | 0 | 0 | 0 | 0 | 0 | 0 | 0 | 0 | 6 |
| + | - | + | 0 | 3 | 2 | 2 | 2 | 0 | 0 | 0 | 0 | 0 | 0 | 0 | 0 | 1 | 0 | 0 | 0 | 0 | 2 | 12 |
| - | + | + | 0 | 0 | 0 | 0 | 0 | 0 | 0 | 0 | 0 | 0 | 0 | 0 | 0 | 0 | 0 | 0 | 0 | 0 | 0 | 0 |
| + | + | + | 0 | 0 | 0 | 0 | 0 | 0 | 0 | 0 | 0 | 0 | 0 | 0 | 0 | 0 | 0 | 0 | 0 | 0 | 0 | 0 |
| **Total** | | | **4** | **5** | **5** | **4** | **4** | **22** | **12** | **8** | **14** | **14** | **4** | **30** | **28** | **35** | **10** | **9** | **11** | **21** | **42** | **282** |
